# Validation of the Termination of Resuscitation Rules in Detroit

**DOI:** 10.7759/cureus.79846

**Published:** 2025-02-28

**Authors:** Arqam Husain, Adam Chalek, Kaab Husain, Ryan J Reece, Robert B Dunne

**Affiliations:** 1 Emergency Medicine, Henry Ford Health System, Detroit, USA; 2 Emergency Medicine, Wayne State University School of Medicine, Detroit, USA; 3 Emergency Medicine, University of Michigan, Flint, USA

**Keywords:** basic life support, cardiac arrest, emergency medical services, out-of-hospital cardiac arrest, termination of resuscitation

## Abstract

Background and objective

The termination of resuscitation (TOR) criteria - which recommends termination when a non-traumatic arrest in an adult is unwitnessed by emergency medical services (EMS), no shocks are administered, and no return of spontaneous circulation (ROSC) occurs - guide physicians in determining the viability of continuing cardiopulmonary resuscitation (CPR) and transporting patients to the hospital. We examined the level of compliance with the current basic life support (BLS) TOR rule and assessed alternative sets of rules to retrospectively derive improved TOR guidelines for out-of-hospital cardiac arrests (OHCA) in Detroit.

Methods

This was a retrospective study involving non-traumatic OHCA cases in Detroit from January 1, 2017, to December 31, 2019, which spans the time frame before and after the BLS TOR rule was officially implemented (June 1, 2018). Data were extracted from the Detroit Cardiac Arrest Registry (DCAR). Patients younger than 18 years of age, those with arrests of traumatic origin, or those with no resuscitation attempted were excluded.

Results

A total of 1,306 individuals were included in our analysis: 656 OHCA cases before the implementation of the BLS TOR rule in Detroit and 650 OHCA after the implementation. BLS TOR criteria were applied to the pre-TOR implementation data with a resulting specificity of 79% (95% CI: 50.7-80.8) and positive predictive value (PPV) of 97.3% (95% CI: 95.5-98.6). Survival to hospital discharge when termination was recommended was projected at 2.9% (13/444). The overall transportation rate was 85% (559/656). Post-TOR implementation, specificity was 88.9% (95% CI: 78.6-99.1) and PPV was 99.1% (95% CI: 98.3-99.9). Survival to hospital discharge was 0.88% (4/453) with a 69% (451/650) overall transportation rate. Post-hoc addition of age or EMS time to the patient side increased transportation rates to 81% (529/650) and 88% (571/650), respectively, and decreased false positive terminations to 0.84% (2/237) and 0% (0/148), respectively.

Conclusions

Overall survival and futile transportation rates decreased when TOR was applied since the implementation of the BLS TOR rule in Detroit. The addition of EMS time to the patient side or patient age to current TOR guidelines suggested improved performance. Although the additional criteria resulted in higher transportation rates, these factors may be useful for physicians to consider when deciding to transport patients. However, further derivation and validation are needed to create optimal TOR guidelines.

## Introduction

Out-of-hospital cardiac arrest (OHCA) is a major public health concern globally, with more than 350,000 OHCAs occurring annually in the United States [1]. Although overall outcomes associated with OHCA are typically poor, survival rates vary significantly across different geographies. Some cities report OHCA as almost uniformly fatal, while others have achieved survival rates as high as 20-40% [2]. These variations are influenced in part by differences in emergency medical services (EMS) response systems, bystander CPR rates, public access to defibrillation, and post-resuscitation care protocols, among other factors.

Despite extensive research, OHCA continues to be a significant public health issue with high mortality rates. Additionally, prognosis remains poor for those resuscitated from an OHCA, with less than half surviving until discharge, and among these survivors, a third suffer from persistent neurologic deficits with less than half returning to normal cognitive function [3,4]. One study revealed that 50% of OHCA survivors exhibit cognitive deficits due to cerebral injury [5]. The initial goal of resuscitative efforts in OHCA is to achieve a return of spontaneous circulation (ROSC), a cornerstone in the chain of survival [6]. A study in the Netherlands has highlighted that survival markedly decreases with delays in ROSC, emphasizing the time-sensitive nature of OHCA and the importance of timely hospital transportation [7]. 

Validation and implementation studies of the basic life support (BLS) termination of resuscitation (TOR) rule

The termination of resuscitation (TOR) criteria were developed in 2002 to aid physicians in determining the viability of continuing cardiopulmonary resuscitation (CPR) and the appropriateness of hospital transport. International basic life support (BLS) guidelines suggest terminating resuscitation if the cardiac arrest was unwitnessed by EMS, if ROSC was not achieved despite efforts, and if the patient had a non-shockable rhythm [8]. Retrospective analyses have shown that adherence to BLS TOR guidelines accurately predicted 100% of fatal outcomes and could reduce the futile transport of patients with fatal outcomes by 40% [9,10]. One report indicated that each additional minute spent on-scene beyond 20 minutes resulted in a 6% decrease in 30-day survival [7].

Futile transport is not consistent with evidence-based practice; moreover, it increases risk and consumes scarce ED resources while reducing the likelihood of survival [11]. A meta-analysis has reported that less than 1% of patients recommended for termination survived, suggesting medical futility [12,13]. However, EMS implementation of BLS TOR guidelines has been inconsistent, often relying solely on ROSC or CPR duration - non-validated measures - making it difficult to confirm the effectiveness of these guidelines [14,15]. Additionally, findings from a recent systematic review and meta-analysis suggested that current TOR guidelines may miss a substantial number of survivors, highlighting the need for potential revisions to current TOR guidelines [11].

Other TOR rules

Since the creation of the BLS TOR guidelines, numerous studies have expanded upon them in the hopes of improving OHCA care. In Korea, incorporating additional rules - such as asystole as the initial rhythm and age over 60 years - increased the positive predictive value (PPV) for poor neurologic outcomes from 99.3% to 99.7-99.9% [16]. In Japan, researchers developed TOR guidelines to guide EMS on immediate resuscitation decisions: these included an unshockable initial rhythm, unwitnessed by bystanders, no prehospital ROSC, or age over 73 years, significantly predicting unfavorable neurological outcomes at one month [17,18]. Specifying the initial rhythm as asystole instead of a non-shockable rhythm improved predictions of long-term unfavorable neurological outcomes [19].

In 2002, the city of Detroit reported that merely 6% of OHCA patients survived till hospital admission and only 0.2% to discharge, exhibiting the nation's lowest survival rates. Targeted efforts to improve the city's chain of survival, especially within EMS, have led to increasing survival rates: from 3.7% in 2014 to 5.4% in 2015, and 6.4% in 2016 - a 73% improvement over three years [20]. The coronavirus disease (2019) COVID-19 pandemic in 2020 mandated protocol changes in Detroit's patient care due to resource constraints and infection control measures, including BLS TOR criteria modifications for OHCA, where patients not achieving ROSC within 10 minutes were considered for termination. Research indicated that despite the more inclusive TOR guidelines, the ROSC rate had not significantly changed [21]. This finding suggests that stricter TOR rules might more effectively predict ROSC and long-term outcomes.

Detroit is the largest city on the US-Canada border, with an estimated population of 672,795 in 2016, 13% of whom are aged 65 years or older. The city has significant socioeconomic challenges: 48.2% owner-occupied housing units, a median household income of $26,249, 39.4% of residents living below the poverty level, and 84% identifying as African-American. There are approximately 130,000 calls for service per year with about 80,000 transports. The Detroit Fire Department operates six non-transporting squads and 27 fire engines, all licensed at the medical first responder (MFR) level. The department also runs 27 BLS ambulances and nine advanced life support (ALS) ambulances, supplemented by eight ambulances from four private companies providing peak-hour coverage. A unique aspect of the EMS system in Detroit is that it is one of the few large systems with primarily BLS responders. "Echo" or "delta" level service calls prompt dispatch of the nearest ambulance and MFR apparatus, and many arrests receive only a BLS response with no ALS intercept or ALS preferential dispatch due to limited ALS units.

Despite recommendations from the American Heart Association and other global health organizations regarding TOR rules, adherence remains low. This not only leads to significant costs on futile care but also strains EMS resources, potentially limiting access for other patients in need [22]. Given the low OHCA survival rates and minimal changes with stricter TOR rules, this study aims to primarily examine the impact of BLS TOR rule implementation in Detroit on survival and transportation rates and to secondarily assess whether alternative criteria such as age and EMS response time could enhance predictive accuracy by better predicting ROSC and long-term survival with neurologic function. This study also aims to both validate existing TOR criteria and generate hypotheses for future guideline optimization, particularly in predominantly BLS systems. Detroit's unique characteristics - including its primarily BLS-based EMS system, majority African-American population, and high poverty rate - make it an important setting for studying TOR guidelines, as existing criteria may perform differently in resource-limited urban environments. 

This article was previously presented as a research abstract at the 2021 American Heart Association's Resuscitation Science Symposium on November 13, 2021.

## Materials and methods

This was a retrospective study involving non-traumatic OHCA cases in Detroit between January 1, 2017, and December 31, 2019, spanning before and after BLS TOR rule implementation in Detroit (June 1, 2018). We gathered information from the Detroit Cardiac Arrest Registry (DCAR), specifically utilizing data from the Cardiac Arrest Registry to Enhance Survival (CARES) database. Patients younger than 18 years of age, those with arrests of traumatic origin, or those with no resuscitation attempted were excluded. The Institutional Review Board (IRB) of Wayne State University reviewed CARES and deemed it exempt. CARES is a comprehensive registry that tracks OHCA data following standardized Utstein-style reporting guidelines. Established in collaboration between the Centers for Disease Control and Prevention (CDC) and Emory University School of Medicine in 2004, CARES includes data from over 1,800 hospitals and 1,400 EMS agencies across 23 states. 

Cardiac arrest care follows standard protocols, and resuscitation attempts are included in Detroit CARES data, including field terminations. BLS protocols involve conducting three CPR cycles (six minutes) on-scene before preparing for transport. If ROSC is not achieved, CPR continues for 30 minutes before consulting on-line medical control for termination decisions. Physician permission is required in the state of Michigan for TOR to be applied. Medical control can also be contacted at any time during the process for further guidance. Utilizing the SafetyPad prehospital electronic health record system, OHCA characteristics were recorded retrospectively. ROSC is defined by the CARES criteria: sustaining a pulse for 20 or more minutes or achieving a pulse before EMS care termination. 

Descriptive statistics compared pre-TOR and post-TOR implementation arrest characteristics. Univariate comparisons for continuous and categorical data were analyzed as mean difference (MD) or odds ratio (OR), respectively, with 95% CI. Additional criteria assessed alongside the BLS TOR rule included the addition of the patient’s age being greater than 64 years and EMS response time being longer than seven minutes and 27 seconds to see whether stricter criteria can help better predict ROSC and long-term survival with neurologic function.

## Results

A total of 1,306 individuals were included in our analysis: 656 OHCA cases before implementing the BLS TOR rule in Detroit and 650 OHCA cases after implementing the BLS TOR rule in Detroit. Table 1 and Table 2 present the demographics and characteristics of patients with non-traumatic OHCA who underwent EMS resuscitation.

**Table 1:** OHCA demographics before and after BLS TOR criteria implementation BLS: basic life support; OHCA: out-of-hospital cardiac arrest; TOR: termination of resuscitation

Variable		Pre-TOR implementation (n=656)	Post-TOR implementation (n=650)
Age, years	Mean	62	62
	Median	63	63
Gender, % (n)	Male	41.2% (270)	56.5% (367)
	Female	58.8% (386)	43.5% (283)
Race/ethnicity, % (n)	Black	86.1% (565)	88.0% (572)
	White	12.7% (83)	9.8% (64)
	Hispanic	0.9% (6)	1.7% (11)
	Native American	0.2% (1)	0.5% (3)
	Asian	0.2% (1)	0% (0)
Comorbidities, % (n)	Hypertension	23.5% (154)	28.6% (186)
	Heart disease	17.2% (113)	18.0% (117)
	Diabetes	13.0% (85)	13.2% (86)
	Respiratory disease	10.4% (68)	9.7% (63)
	Renal disease	8.1% (53)	7.2% (47)
	Stroke	3.2% (21)	3.5% (23)

**Table 2:** OHCA characteristics before and after BLS TOR criteria implementation BLS: basic life support; CPC: Cerebral Performance Category; CPR: cardiopulmonary resuscitation; ED: emergency department; EMS: emergency medical services; OHCA: out-of-hospital cardiac arrest; ROSC: return of spontaneous circulation; TOR: termination of resuscitation

Characteristic		Pre-TOR implementation (n=656)	Post-TOR implementation (n=650)
BLS initial rhythm, n	Unknown unshockable	562	567
	Unknown shockable	94	83
EMS response time	25th percentile, min	04:16	04:26
	50th percentile, min	05:50	06:00
	75th percentile, min	07:50	08:14
	Mean, min	06:23	06:34
	Median, min	05:50	06:00
	EMS response time >5:51 minutes (n)	325	315
	EMS response time >7:51 minutes (n)	162	162
Witnessed, n	Unwitnessed by EMS	578	573
	Arrest witnessed by EMS	78	77
Bystander CPR, n	Yes	253	266
	No	403	384
Arrest location, n	Home/residence	451	453
	Nursing home	116	100
	Public place	26	42
ROSC obtained, n	Yes	74	84
	No	582	570
End of the event, n	Pronounced dead in ED	171	141
	Dead in field	105	211
	Ongoing resuscitation in ED	380	298
Death, n	Overall deaths	618	614
	Deaths after admission	120	94
Survival, n	At discharge	38	36
CPC score of total survivors, n	1 or 2	16	16
	>3	21	20

BLS TOR criteria were applied to the pre-TOR implementation data, resulting in a specificity of 65.8% (95% CI: 50.7-80.8) and PPV of 97.3% (95% CI: 95.5-98.6). The overall transportation rate was 83.9% (551/656) while the transportation rate when TOR was met was 77% (342/444). The overall proportion of patients admitted to the hospital was 24.1% (158/656) while that admitted to the hospital when termination was recommended was 29.5% (131/444). The overall survival rate was 5.8% (38/656). Survival to hospital discharge when termination was recommended was projected at 2.9% (13/444). The proportion of patients discharged with overall good neurological function defined as a Cerebral Performance Category (CPC) score of 1 or 2 was 2.4% (16/656) whereas that of patients discharged alive with good neurological function when the BLS TOR rule was met was 0.9% (4/444).

**Figure 1:**
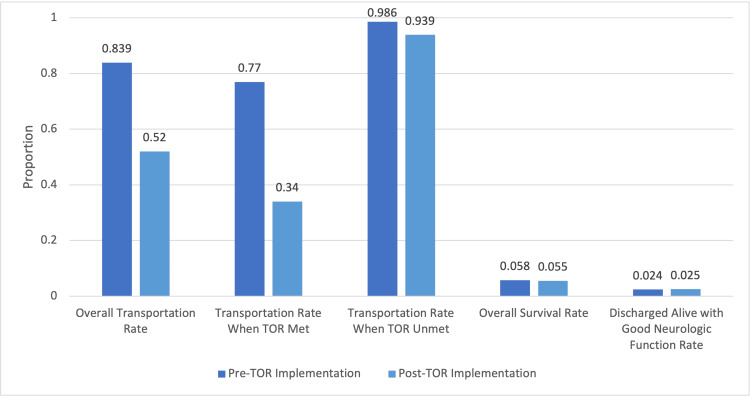
Pre-TOR and post-TOR implementation effect on outcomes in patients by proportion (%) TOR: termination of resuscitation

Official implementation of the BLS TOR rule caused transportation rates to decrease without affecting survival. Post-TOR implementation, specificity was 88.9% (95% CI: 78.6-99.1) and PPV was 99.1% (95% CI: 98.3-99.9). The overall transportation rate was 52% (339/650) while the transportation rate when TOR was met was 34% (154/453). The overall percentage of patients admitted to the hospital was 20% (130/650) while the percentage of patients admitted to the hospital when termination was recommended was 10.2% (46/453). The overall survival rate was 5.5% (36/650). Survival to hospital discharge when termination was recommended was 0.88% (4/453). The proportion of patients discharged with overall good neurological function was 2.5% (16/650) whereas that of patients discharged alive with good neurological function when the BLS TOR rule was met was 0% (0/453). These findings are presented in Table 3 and Figure 1.

**Table 3:** Statistical analysis of OHCA before and after BLS TOR criteria implementation BLS: basic life support; CI: confidence interval; OHCA: out-of-hospital cardiac arrest; TOR: termination of resuscitation

	Pre-TOR implementation	Post-TOR implementation
Survival rate when termination recommended by TOR	2.9%	0.9%
Survival rate when termination not recommended by TOR	11.8%	16.2%
Overall survival rate	5.8%	5.5%
Percentage transported when TOR was met	77.0%	34.0%
Sensitivity % (95% CI)	69.7% (66.1-73.4)	73.1% (69.6-76.6)
Specificity % (95% CI)	65.8% (50.7-80.8)	88.9% (78.6-99.1)
Positive predictive value % (95% CI)	97.1% (95.5-98.6)	99.1% (98.3-99.9)
Negative predictive value % (95% CI)	11.8% (7.4-16.1)	16.2% (11.1-21.4)
False positive rate	34.2%	11.1%
Pre-test probability	94.2%	94.5%
Post-test probability	97.0%	99.1%

**Figure 2:**
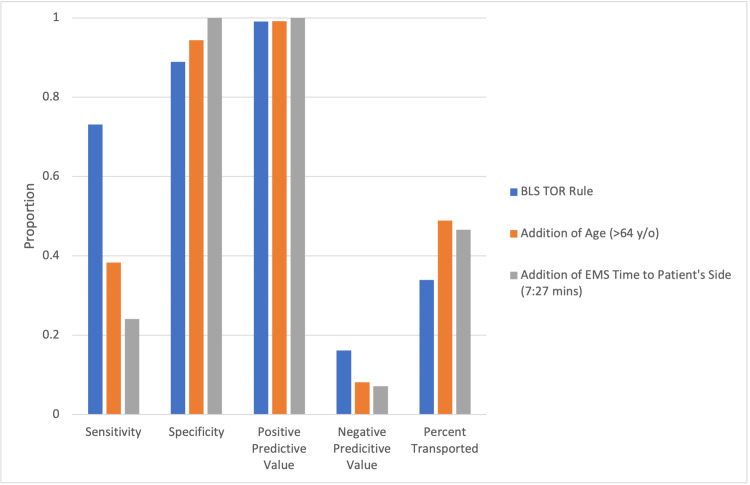
Impact of post-hoc addition of patient age and EMS time to patient side in BLS TOR rule BLS: basic life support; EMS: emergency medical services; TOR: termination of resuscitation

Post-hoc addition of age or EMS time to the patient side increased specificity from 88.9% to 94.4% (34/36) and 100% (36/36), respectively, while maintaining PPV from 99.1% (449/453) to 99.2% (235/237) and 100% (148/148), respectively. Additionally, the added criteria of age or EMS time to the patient side increased transportation rates from 34% to 49% (116/237) and 47% (69/148), respectively, while false positive terminations (patients who survived when BLS TOR rule was met) decreased slightly from 0.88% (4/453) to 0.84% (2/237) and 0% (0/148), respectively. The proportion of patients discharged alive with good neurological function when the BLS TOR rule was met was 0% in both cases. These findings are displayed in Figure 2.

## Discussion

The city of Detroit is one of the few large systems that has primarily BLS responders. Due to the paucity of ALS units, many OHCA cases receive only a BLS response with no ALS intercept or ALS preferential dispatch. Official implementation of BLS TOR criteria in Detroit substantially decreased transportation rates for OHCA patients without affecting survival rates. The transportation rate fell from 83.9% pre-TOR to 52% post-TOR, a 38% decrease. Despite fewer transportations, the overall survival rate remained relatively constant, decreasing slightly from 5.8% to 5.5%. 

When determining OHCA patient transportation, it is crucial to weigh the benefits against the costs and potential futility. Outcomes for OHCA patients are generally poor, with one meta-analysis reporting 22% survival to hospital admission, 8.8% survival to discharge, 10.7% one-month survival, and 7.7% one-year survival [23]. Reducing transportation rates and employing selective criteria can substantially decrease medical costs. Our study indicates that post-TOR implementation, 20% of patients survived to admission, 5.5% to discharge, and 2.5% exhibited good neurological function. Among patients meeting BLS TOR criteria but still transported, only 10% survived to admission, and 0.9% survived to discharge with no patient showing good neurological function. In the state of Michigan, the decision to terminate resuscitation for BLS or ALS requires a physician’s decision, and some physicians do not feel comfortable terminating resuscitations despite clearly meeting BLS TOR criteria. Strict adherence to BLS TOR criteria could therefore curtail unnecessary transportation. Enhancing transport specificity through improved TOR guidelines will help avoid futile transports and save significant time and resources.

Studies attempting to improve TOR specificity include one that allowed pre-hospital termination for adult cardiac arrests of presumed cardiac etiology without a shockable rhythm and no ROSC on-scene. This guideline identified 832 cases for termination, with only 0.4% survival, showing high specificity (99.1%) and a lower transportation rate (60.7%). However, the sensitivity was low (46.5%), indicating potential missed survival cases [24]. Such guidelines significantly improve cost-effectiveness but might miss survival cases. Findings from a recent systematic review and meta-analysis suggested that current TOR guidelines may miss a substantial number of survivors and that the evidence has low certainty to ascertain who will die and who will survive, thus highlighting the need for potential revisions to current TOR guidelines [11].

The decision to transport OHCA patients is fraught with considerations and critical in determining survival chances. One study showed that longer transportation time intervals (TTI) resulted in lower chances of good neurologic recovery: a TTI of four minutes or less had a 1.0% chance, while a TTI of 12 minutes or more had only a 0.5% chance [25]. When considering updates to the BLS TOR rule, our data shows that adding EMS time to the patient’s side to TOR guidelines decreased false positive terminations slightly and maintained low GNF discharge rates for patients meeting these criteria.

These findings have significant implications for EMS providers and policymakers. While BLS TOR criteria reduce unnecessary transports and healthcare burdens, ensuring patients who can benefit from transport aren't denied is crucial. However, the decision to increase transportation rates through modified TOR criteria must be weighed against system-level impacts. Higher transportation rates can strain EMS resources, increase emergency department crowding, and divert resources from other time-sensitive emergencies. In resource-limited settings, additional OHCA transportations require careful consideration of these opportunity costs. While our findings suggest potential benefits from considering age and EMS response time in transportation decisions, the optimal balance between identifying potential survivors and avoiding futile transports may vary across different EMS systems.

Detroit's OHCA survival rates remain lower than the national average both before and after TOR implementation, underscoring the need for continuous improvement efforts. However, the generalizability of these findings requires careful consideration. Systems with predominantly ALS response, different geographic characteristics, or more abundant hospital resources may find different optimal thresholds for TOR criteria. Additionally, communities with different demographic profiles or higher baseline survival rates may need to adjust these criteria to their local context. Evaluating TOR criteria effectiveness across various contexts and refining inclusion criteria can enhance their applicability and reliability.

An important consideration is the generalizability of these findings to other settings. While the study focused on OHCA patients in Detroit, it is unclear whether the results would be the same in other settings with varying EMS practices and sufficiently different socioeconomic, demographic, geographic, and population health characteristics than in Detroit. Further research is needed to evaluate the effectiveness of BLS TOR criteria in different contexts and to identify factors that may influence transportation and survival rates. Additionally, retrospective, registry-based data has inherent limitations such as potential data entry errors, missing information, and reporting biases from organizations providing data to CARES. Although reporting bias and missing data can be problematic for large databases, we observed that the CARES database had no unanswered fields, and CARES provides clear, objective definitions for data recording to minimize potential biases.

## Conclusions

Overall survival when TOR was recommended and futile transportation rates decreased since implementing BLS TOR guidelines in Detroit. The addition of EMS time to the patient side or the patient’s age to the current TOR guidelines suggested improved performance. Although the additional criteria resulted in higher transportation rates, these factors may help physicians in deciding OHCA patient transport. Terminating resuscitation requires further derivation and validation studies to optimize TOR guidelines for OHCA.
